# Integrating nutrition into health systems: What the evidence advocates

**DOI:** 10.1111/mcn.12738

**Published:** 2019-02-12

**Authors:** Rehana A. Salam, Jai K. Das, Zulfiqar A. Bhutta

**Affiliations:** ^1^ Division of Woman and Child Health The Aga Khan University Karachi Pakistan; ^2^ South Australian Health and Medical Research Institute; and University of Adelaide Adelaide Australia; ^3^ Centre for Global Child Health The Hospital for Sick Children Toronto Ontario Canada; ^4^ Centre of Excellence in Women and Child Health The Aga Khan University Karachi Pakistan

**Keywords:** health integration, health system, integration, nutrition, nutrition programmes, nutrition specific

## Abstract

There is considerable evidence of positive health and nutrition outcomes resulting from integrating nutrition‐specific interventions into health systems; however, current knowledge on establishing and sustaining effective integration of nutrition into health systems is limited. The objective of this review is to map the existing types of integration platforms and review the evidence on integrated health and nutrition programmes' impacts on specific nutrition outcomes. A literature search was conducted, and integrated nutrition programmes were examined through the lens of the six World Health Organization (WHO) building blocks, including the demand side. Forty‐five studies were included in this review, outlining the integration of nutrition‐specific interventions with various programmes, including integrated community case management and Integrated Management of Childhood Illness, Child Health Days, immunization, early child development, and cash transfers. Limited quantitative data were suggestive of some positive impact on nutrition and non‐nutrition outcomes with no adverse effects on primary programme delivery. Through the lens of the six WHO building blocks, service delivery and health workforce were found to be well‐integrated, but governance, information systems, finance and supplies and technology were less well‐integrated. Integrating nutrition‐specific interventions into health systems may ensure efficient service delivery while having an impact on nutrition outcomes. There is no single successful model of integration; it varies according to the context and demands of the particular setting in which integration occurs. There is a need for more well‐planned programmes considering all the health systems building blocks to ensure compliance and sustainability.

**Key messages**

- Scaling‐up nutrition interventions and integrated delivery of nutrition‐specific intervention into existing successful health programmes could offer an opportunity, especially in low‐ and middle‐income countries.
- Current evidence underscores the opportunity for the integrated nutrition delivery strategies, frequently at the point of service delivery, to tackle the burden of malnutrition.
- Meticulous planning is required to design programmes, taking into account various context‐specific contextual factors to ensure compliance, impact, and sustainability.

## 1 INTRODUCTION

Globally, policymakers and implementation bodies need to put in concerted effort to explore innovative means to reduce the existing high burden of malnutrition and to ensure the achievement of the Sustainable Development Goals 2 and 3, namely, to end hunger, achieve food security and improve nutrition, promote sustainable agriculture, ensure healthy lives, and promote well‐being for all ages (Horton et al., 2013; World Health Organization [WHO], 2015). Malnutrition currently affects one in three persons globally, with undernutrition as the underlying cause of nearly half (45%) of all deaths among children under the age of 5 years and contributing to 20% of maternal mortality (Black et al., 2013). An estimated 2 billion people experience deficiencies in essential vitamins and minerals, resulting in 155 million children who are stunted and 52 million who are wasted (Hawkes & Fanzo, 2017).

Scaling up several evidence‐based interventions could significantly impact nutritional status, especially in low‐ and middle‐income countries. However, vertically scaling up the coverage of existing interventions in isolation will not suffice unless as a temporary measure or a rapid response (R. A. Atun, Bennett, Duran, & WHO, 2008); therefore, integrating these nutrition interventions into existing health system programmes has to be further explored (Bhutta et al., 2013). The literature has proposed various definitions of integration, differing according to the context and ranging from a package of preventive and curative health interventions to multipurpose service‐delivery points (R. Atun, de Jongh, Secci, Ohiri, & Adeyi, 2010; Berer, 2003; Briggs & Garner, 2006; Contandriopoulos, Denis, Nassera, & Rodríguez, 2003; Kodner & Spreeuwenberg, 2002; Lynne, Michael, Lisa, Patti, & Audrey, n.d.; WHO, 2008). There is considerable evidence of positive health and nutrition outcomes from integrated nutrition interventions (Aguayo et al., 2013; Amadi, Imikendu, Sakala, Banda, & Kelly, 2016; Brits et al., 2017; Masanja, Schellenberg, De Savigny, Mshinda, & Victora, 2005; Schellenberg et al., 2004; Tadesse, Worku, Berhane, & Ekström, 2017), but due to the complexity of health programmes and the contexts in which they operate, it is difficult to establish which points of integration are the most effective. Numerous integrated health initiatives have arisen over the last few years, for example, the integrated Global Action Plan for the Prevention and Control of Pneumonia and Diarrhoea (WHO‐UNICEF, 2013), integrated community case management (iCCM; Young, Wolfheim, Marsh, & Hammamy, 2012), Integrated Management of Childhood Illness (IMCI; WHO‐UNICEF, 1997), and Research on Food Assistance for Nutritional Impact (Fenn et al., 2017). There has been significant interest globally in making more nutrition‐sensitive investments in related sectors (e.g., agriculture, social safety nets, early child development [ECD], classroom education, and water, sanitation, and hygiene) and utilizing these platforms for integrating and expanding delivery of nutrition‐specific interventions (Gillespie et al., 2013; Ruel, Alderman, & Maternal & Child Nutrition Study Group, 2013).

However, current knowledge and guidance on establishing and sustaining effective integration even between health and nutrition sectors are limited (Armitage, Suter, Oelke, & Adair, 2009; R. Atun et al., 2010; R. A. Atun et al., 2008). The focus has largely been on which interventions are evidence based, with insufficient attention to scale‐up, equity, and integration within health systems. There are missed opportunities for scaling up nutrition‐specific interventions through strengthening the existing health system with a nutrition lens and through improving quality of care. The objective of this scoping review was to map the existing integration platforms, describe an innovative conceptual framework, and review the evidence on integrated health and nutrition programmes and their impacts on specific nutrition outcomes.

## 2 METHODS

We followed the PRISMA statement (Preferred Reporting Items for Systematic reviews and Meta‐Analyses) for the conduct and reporting of this review. We conducted a search for relevant literature in electronic databases, including MEDLINE, PubMed, and CENTRAL. Our search strategy utilized medical subject heading terms and free text terms, identified through electronic reference libraries of indexed medical journals and analytical reviews, and major search terms included the following: integrated delivery of health care, comprehensive health care, integrated programmes, primary health care, nutrition programmes, maternal nutrition, child nutrition, maternal nutrition, and undernutrition. Our last search date was October 15, 2017. Two abstractors screened titles, abstracts, and full texts through the Covidence® screening and extraction tool to identify relevant studies. A third reviewer resolved any disagreements on the selection of studies.

Inclusion criteria: We included only peer‐reviewed publications evaluating programmes integrating nutrition‐specific interventions with other programmes without any date restrictions. We defined nutrition integration as “the extent of adoption and eventual assimilation of nutrition interventions into critical health system functions (building blocks).”

Exclusion criteria: We excluded studies evaluating the impact of stand‐alone programmes on nutrition outcomes. We also excluded studies evaluating the impact of packaged delivery of interventions in which nutrition interventions were a part of the package as it did not integrate nutrition interventions into existing health systems and did not follow our definition of integration. We extracted data from studies on the following parameters: author/year, study design, study setting, primary programme details, integrated programme details, control group (if any), year and duration of implementation, target population, integration component details, gender‐equity indicators, nutrition‐specific outcomes, other programme‐specific outcomes, quality assessment indicators, and programme enablers and barriers.

After extracting the included studies, we mapped the integrated programmes based on the primary programmes, assessed the extent of integration in the identified six World Health Organization (WHO) building blocks, and analysed the quantitative impact on nutrition and nonnutrition outcomes. We defined the “primary programme” as an existing programme/platform integrating nutrition‐specific interventions. We mapped integrated nutrition‐specific interventions according to the primary platform (defined as the primary programmes into which nutrition‐specific interventions were integrated). We described the extent of integration among all the six building blocks (leadership/governance, financing, health information systems, health workforce, supplies and technology, and service delivery) individually for each paper included in this review, along with the demand‐side platforms. We ascribed a score from 0 to 3 to the extent of integration on the six domains (WHO, 2010), and the criterion for these ratings is detailed in Table 1. For all categories mapped, we calculated mean scores for each building block and graphically depicted the integration through spider web charts. Two authors independently scored, and consensus was achieved through discussion and involvement of third author in case of any discrepancy.

**Table 1:** Scoring extent of integration in each building block

**Building blocks**	Degree of integration		
	1 = not integrated	2 = partially integrated	3 = fully integrated
**Governance**	Complete governance of the nutrition‐specific interventions is under an independent body other than the primary programme	Nutrition‐specific interventions' governance is shared with the primary programme governance	Complete governance of the nutrition‐specific interventions is under the primary programme
**Financing**	Finances provided solely by an entity separate from the primary programme	Sharing of finances between the primary programme and the nutrition‐specific interventions	All the financial requirements are met through the primary programme
**Information systems**	The nutrition‐specific interventions have separate data procedures, rather than being included in the primary programmes	Nutrition‐specific interventions have separate data procedures, in addition to being somehow included in existing procedures for the primary programme	Data collection for the nutrition‐specific interventions is through existing primary programmes mechanisms
**Health workforce**	Additional staff carry out the nutrition‐specific interventions, parallel to the primary programme staff	Existing staff and additional staff jointly carry out the interventions of the primary programme and the nutrition‐specific interventions	The existing staff of the primary programme performed the entire duties of the nutrition‐specific interventions
**Supplies and Technology**	The nutrition‐specific interventions have separate logistics and distribution support, separate from the primary programmes	Nutrition‐specific interventions use existing logistic and distribution support, along with their own new channels	Existing distribution channels are used for the delivery of the nutrition‐specific interventions
**Service delivery**	Nutrition‐specific interventions have service delivery centres or mode of delivery separate from the primary programme	Nutrition‐specific interventions partially carried out through the existing primary programmes service delivery mechanisms	All the nutrition‐specific interventions are delivered through the primary programme channel

Where available, we extracted quantitative data for nutrition‐specific and primary programme outcomes. When data were available from more than one study for any outcome, we conducted meta‐analysis using Review Manager Software Version 5.3. We reported effect estimates as either risk ratio (RR) or odds ratio (OR) with 95% confidence intervals (CI). We presented the meta‐analysis as forest plots depicting the individual study estimates as well as the pooled estimates. Where we could not pool the outcomes through meta‐analysis, we provided a descriptive analysis. We also attempted to determine whether integrated nutrition interventions reduced gender‐equity disparities (sex, age, disability, poverty).

We assessed the quality of the included studies using the Cochrane risk of bias assessment tool for the randomized controlled trials and quasi‐randomized controlled trials (Higgins, Altman, & Sterne, 2011). For non‐randomized studies, we used the Cochrane Effective Practice and Organization of Care guidelines (EPOC, 2017). We did not assess the quality of the cross‐sectional studies, descriptive studies, and qualitative studies included in the review.

## 3 RESULTS

Conceptual framework: The WHO (2010) has proposed a framework describing health systems in terms of six building blocks: leadership/governance, financing, health information systems, health workforce, supplies and technology, and service delivery. To appreciate the complexities of integrating into health systems, we built our framework—that of integrating nutrition into existing health systems—around these building blocks, alongside the critical aspect of the demand side and community engagement.

Data S1 displays our conceptual framework. We have described all the WHO building blocks across the central‐planning, district, and service‐delivery levels with regard to the integration of nutrition interventions into the health systems. Governance and financing are critical in assessing the degree to which evidence‐based nutrition‐specific interventions are integrated into existing health policies and strategies, as well as the degree to which funds for nutrition‐specific interventions are allocated through existing programmes. Information systems and health workforce help assess how well existing information systems integrate nutrition status and coverage of nutrition‐specific services, as well as whether facility and community health workers (CHWs) are available to offer nutrition‐specific services. The components of supplies, technology, and service delivery help assess how well existing infrastructure and commodities are used for nutrition‐specific interventions, as well as the degree to which health facilities and CHWs are providing quality nutrition‐specific services.

Search results: We identified 13,843 titles in our search and after abstract and full‐text screening and cross referencing; we included 45 papers that met the inclusion criteria. Studies ranged from randomized, controlled trials to qualitative studies (Aguayo et al., 2013; Amadi et al., 2016; Anand, Luman, & O'Connor, 2012; Arifeen et al., 2009; Armstrong et al., 2004; Baqui et al., 2008; Berti, Mildon, Siekmans, Main, & MacDonald, 2010; Bhandari, Mazumder, Taneja, Sommerfelt, & Strand, 2012; Brits et al., 2017; Bryce et al., 2005; Ching, Birmingham, Goodman, Sutter, & Loevinsohn, 2000; Deconinck et al., 2016; Doherty et al., 2010; El Arifeen et al., 2004; Fagerli et al., 2017; Fernandez‐Rao et al., 2014; Friedman & WoLFheim, 2014; Gowani, Yousafzai, Armstrong, & Bhutta, 2014; Grellety et al., 2017; Grossmann et al., 2015; Guyon et al., 2009; Hodges et al., 2015; Klemm, Villate, Tuazon‐Lopez, & Ramos, 1996; Kouam et al., 2014; Masanja et al., 2005; Mazumder et al., 2014; Miller et al., 2014; Nguyen et al., 2017; Palmer, Diaz, Noordam, & Dalmiya, 2013; Parikh et al., 2010; Puett, Alderman, Sadler, & Coates, 2015; Puett, Coates, Alderman, & Sadler, 2013; Rasanathan et al., 2014; Ropero‐Álvarez, Kurtis, Danovaro‐Holliday, Ruiz‐Matus, & Tambini, 2012; Sadler, Puett, Mothabbir, & Myatt, 2011; Saiyed & Seshadri, 2000; Schellenberg et al., 2004; Singh et al., 2017; Sivanesan, Kumar, Kulkarni, Kamath, & Shetty, 2016; Tadesse et al., 2017; Tandon, 1989; Taneja et al., 2015; Yousafzai, Rasheed, Rizvi, Armstrong, & Bhutta, 2014). Data S1, Figure S1 depicts the search flow diagram, and the quality of the included studies are described in Data S1, Figure S2. Most of the included studies were at high risk of bias for randomization due to inadequate sequence generation and allocation concealment, as well as the lack of blinding of the participants and personnel and blinding of the outcome assessor. Blinding could not be achieved due to the nature of the intervention.

### 3.1 Mapping nutrition integration based on the primary programmes

We reviewed and mapped all studies according to the primary programmes into which nutrition‐specific interventions were integrated. These primary programmes, or “integration platforms,” included integrating nutrition into Integrated Management of Childhood Illness and integrated community case management (IMCI/iCCM), integrating management of severe and moderate acute malnutrition (SAM/MAM) into health services, integrating nutrition into Child Health Days (CHD) and integrating nutrition into immunization, as well as integrating nutrition into social programmes, including ECD and cash transfers. We combined the few studies that could not be categorized in the above categories and were classified as “other programmes;” these programmes integrated nutrition‐specific interventions, including promotion of breastfeeding and appropriate complementary feeding, feeding practices, growth monitoring, supplementary nutrition, vitamin A supplementation, home fortification, screening and management for malnutrition into existing community health setups, and maternal, newborn, and child health centres and clinics. Data S1, Table S1 provides the summary of the studies and programmes found for each platform, which were integrated into primary health systems.

### 3.2 Extent of integration in the identified six building blocks

**Figure 1:**
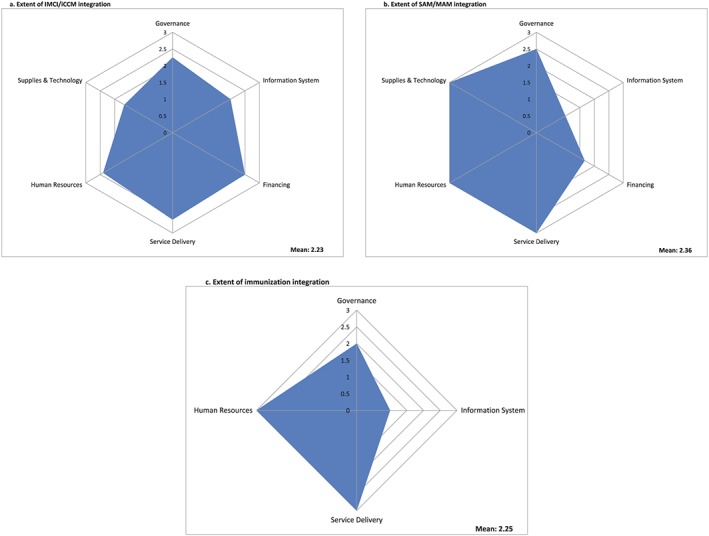
Extent of integration of nutrition in each building block by primary programme

Figure 1 depicts each platform's extent of integration across all building blocks for IMCI/iCCM, SAM/MAM, and immunization. We have not included the spider plots for ECD, cash transfers, and CHD because there were either one or two studies in these domains, and hence, we have only provided a narrative synthesis. In summary, integration of nutrition into IMCI/iCCM was strong, with a mean integration score of 2.23 across the six building blocks. For SAM/MAM into health services, the mean score of integration was 2.36 across all building blocks, with governance, service delivery, health workforce and supplies, and technology all almost fully integrated, whereas information systems and financing were not integrated. For nutrition into immunization, the mean score was 2.25, with service delivery and health workforce fully integrated, whereas governance and information systems were not integrated, and there was no information available from any of the included studies on the finance and supplies and technology. For nutrition into CHD, service delivery and health workforce were fully integrated, but governance, information systems, financing and supplies, and technology were not integrated. For nutrition into ECD, service delivery, health workforce, and information systems were fully integrated, whereas financing and supplies and technology were not integrated, and no information was available from any of the included studies on governance. For nutrition into cash transfer programmes, the one programme included showed service delivery and health workforce being fully integrated, whereas governance, financing and supplies, and technology were not integrated, and no information was available on information systems. We did not objectively rate the building blocks for the “other” category as the primary programmes were disparate.

### 3.3 Quantitative impact on nutrition and non‐nutrition outcomes

There were limited quantitative data, but where they were available, we performed a quantitative analysis for outcomes of the primary programme (nonnutrition‐specific outcomes) and the integrated nutrition intervention (nutrition‐specific outcomes). There were no data in the included studies to perform the gender‐equity analysis. Two platforms (CHD and ECD programmes) did not have sufficient data for quantitative analysis of outcomes. Table 2 summarizes the estimates for the pooled outcomes reported.

**Table 2:** Quantitative impact of integrated nutrition programmes

Outcomes	Pooled effect sizes [RR and 95% CIs]
Integrated nutrition and IMCI/iCCM programmes	
Child younger than 6 months exclusively breastfed	RR: 1.27 [0.70, 2.30]; three studies *I* ^2^ = 98%; random model
Child aged 6–9 months receiving breast milk and complementary feeding	RR: 1.24 [0.56, 2.71]; two studies; *I* ^2^ = 100%; random model
Wasting in children aged 0–23 months (<−2 WHZ)	RR: 1.08 [0.93, 1.24]; three studies; *I* ^2^ = 32%; fixed model
Stunting in children aged 24–59 months	RR: 1.04 [0.97, 1.11]; two studies; I^2^ = 0%; fixed model
Care seeking for children with danger signs	**RR: 1.44 [1.18, 1.75]; three studies; *I*** ^**2**^ **= 76%; random model**
Child illness correctly classified	RR: 6.48 [0.19, 223.87]; two studies; *I* ^2^ = 97%; random model
Child with pneumonia correctly treated	**RR: 2.65 [1.17, 6.02]; three studies; *I*** ^**2**^ **= 79%; random model**
Integrated nutrition and immunization programmes	
Initiated breastfeeding within first hour	**RR: 3.74 [1.21, 11.62]; two studies; *I*** ^**2**^ **= 99%; random model**
Underweight	**RR: 0.47 [0.13, 1.69]; three studies; *I*** ^**2**^ **= 89%; random model**

*Note*. RR: risk ration; WHZ: weight for height *z* score. Bold values indicate statistically significant estimates.

**Figure 2:**
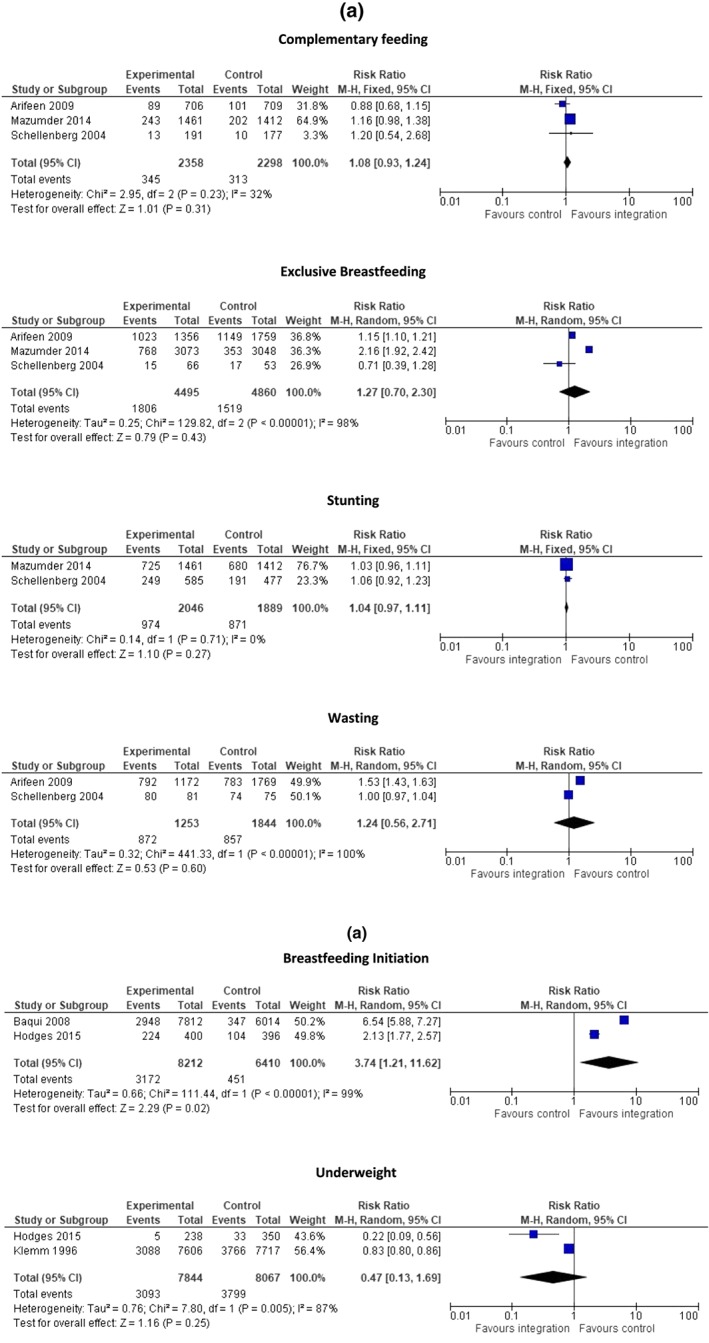
Forest plots for the pooled outcomes for integrating nutrition into IMCI/iCCM Programmes (a) and into immunization programmes (b)

For the integrated nutrition and IMCI/iCCM programmes, pooled analysis of nutrition‐specific outcomes suggests that there was no statistically significant difference in exclusive breastfeeding rates, complementary feeding, or prevalence of wasting and stunting between the integrated programmes and the control group (Figure 2). Descriptive analysis of two studies shows that the proportion of underweight children did not differ significantly where nutrition was integrated into IMCI/iCCM programmes compared with control (Masanja et al., 2005; Schellenberg et al., 2004). In Bangladesh, correct classification of very low weight was significantly higher in the intervention group compared with the control group (83 vs. 0%) (Arifeen et al., 2009). Among pooled non‐nutrition outcomes, integrated nutrition and IMCI/iCCM programmes significantly improved care seeking for children with danger signs (RR: 1.44, 95% CI [1.18, 1.75]; three studies; Arifeen et al., 2009; Mazumder et al., 2014; Schellenberg et al., 2004) and proportion of children with pneumonia correctly treated (RR: 2.65, 95% CI [1.17, 6.02]; three studies) (Arifeen et al., 2009; Bryce et al., 2005; Schellenberg et al., 2004). There was no impact on correct classification of the sick child. Descriptive analysis shows that the Bangladesh programme demonstrated significantly improved correct management of any illness in children (64 vs. 10%) in the intervention group compared with control (Arifeen et al., 2009). The India programme demonstrated significantly reduced infant mortality in the intervention group compared with the control (hazard ratio [HR]: 0.85 [0.77, 0.94]) with no impact on neonatal mortality (Bhandari et al., 2012). Diphtheria, pertussis, tetanus, and measles vaccine coverage also did not differ significantly in the intervention and control groups (Masanja et al., 2005; Mazumder et al., 2014).

Pooled analysis suggests that the integrated nutrition and immunization programmes led to significant increase in early initiation of breastfeeding (RR: 3.74, 95% CI [1.21, 11.62]; two studies) (Baqui et al., 2008; Hodges et al., 2015) but had no impact on underweight (RR: 0.47; 95% CI [0.13, 1.69]; Hodges et al., 2015; Klemm et al., 1996; Figure 2). Descriptive analysis shows that the Sierra Leone programme demonstrated a higher proportion of exclusive breastfeeding rates in the intervention compared with the control group (Data S1; Hodges et al., 2015). Descriptive analysis suggests that the programme from the Philippines demonstrated higher vitamin A capsule coverage and lower night blindness (OR: 0.40, [0.20, 0.78]) in the intervention group as compared with the control (Baqui et al., 2008; Klemm et al., 1996).

For integrated SAM/MAM programmes, we could not conduct a meta‐analysis for any of the nutrition‐specific or non‐nutrition outcomes because all the studies were one‐time cross‐sectional surveys and did not provide data for comparison. Recovery from SAM ranged from 18% in a facility‐based management programme in India to 23% in the primary care health care system in Ethiopia, 50% in South Africa, 65% in the community component in India, and 70% in Zambia (Aguayo et al., 2013; Amadi et al., 2016; Brits et al., 2017; Tadesse et al., 2017). In the integrated Zambia programme, recovery from MAM was demonstrated to be around 80%, and there was significant impact on SAM case fatality rates (RR: 10.9 [3.4, 34.8]) (Amadi et al., 2016).

A single study on integrated nutrition and cash transfer programmes (Grellety et al., 2017) reported significantly higher SAM recovery (HR: 1.35, 95% CI [1.10, 1.69]), lower MAM relapse (HR: 0.21, 95% CI [0.11, 0.41]), and lower SAM relapse (HR: 0.30, 95% CI [0.16, 0.58]) in the integrated group compared with the control group. Change in weight, weight for age *z* score, weight for height *z* score, and body mass index *z* score were also significantly better in the intervention group compared with the control group. There was no difference in change in height/length, height/age, or mid‐upper arm circumference between intervention and control groups.

For integrated nutrition and other programmes (programmes that could not be categorized in the above categories and integrated nutrition‐specific interventions, including promotion of breastfeeding and appropriate complementary feeding, feeding practices, growth monitoring, supplementary nutrition, vitamin A supplementation, home fortification, screening and management for malnutrition into existing community health setups, and maternal, newborn, and child health centres and clinics), we could not pool any of the outcomes. Among nutrition‐specific outcomes, the India programme showed significantly improved early initiation of breastfeeding (OR: 2.04, 95% CI [1.20, 3.45]) and exclusive breastfeeding (OR: 0.61, 95% CI [0.36, 1.03]; Singh et al., 2017), and programmes for Kenya and Bangladesh suggested significantly higher intervention coverage for vitamin A supplementation, paediatric iron folic acid supplementation, and supplementary nutrition (Fagerli et al., 2017; Nguyen et al., 2017). The Kenya programme also reported significant increase in the exclusive breastfeeding rates from baseline to end line, as well as improved antenatal visits, health facility delivery, and postnatal visits (Fagerli et al., 2017).

## 4 DISCUSSION

Our scoping review revealed a general paucity of detailed information on the integration of nutrition interventions within the health sector and the similarly aligned social sector. The majority of nutrition‐specific interventions are dependent on health systems for their successful delivery, but the coverage of nutrition interventions in many developing countries is low and sometimes not part of the essential package of services. There is a general lack of a global consensus on an agreed definition, framework, and minimum standards for integrating nutrition‐specific interventions into health service delivery. Hence, our conceptual framework revolved around the core health system building blocks and how existing nutrition interventions are assimilated around these critical health system functions.

We found various nutrition‐specific interventions integrated with various programmes, including IMCI/iCCM, CHD, immunization, ECD, and cash transfers. In analysing the extent of integration, the programmes reviewed were often well‐integrated in the building blocks of service delivery and health workforce, given that most utilized existing modes of service delivery and existing staff to deliver the services. Governance was also one of the well‐integrated building blocks because the majority of programmes involved incorporating nutrition‐specific interventions into the existing health strategies and policies to ensure future sustainability. In contrast, information systems, financing and supplies/technologies were the least integrated building blocks, and most programmes relied on a separate channel for these.

We could not pool data for all available outcomes given that different programmes had used different outcome measures, and the analytical methods were not rigorous. There were also no data to carry out gender‐equity analysis, a critical consideration for integrated programmes presently. There were, however, several important findings from the limited information suggesting that integrated nutrition interventions were associated with a significant increase in breastfeeding initiation rates, improved recovery, and reduced relapse of children with SAM and MAM and improved coverage of vitamin A supplementation. Effects on other outcomes such as exclusive breastfeeding, stunting, wasting, and underweight were non‐significant. Findings also suggested that integrated nutrition programmes could have a positive impact on the primary programme. To illustrate, integration of nutrition into IMCI platforms led to significant improvements in care seeking for danger signs and correct pneumonia treatment, and individual programmes also reported improved coverage with antenatal and postnatal care, facility delivery, and vaccines.

Table 3 summarises the building blocks of integration and its specific enablers and barriers. Among the integrated nutrition programmes reviewed, service delivery and health workforce were apparently well‐integrated, although several reports highlighted issues with the deployment, supervision, motivation, and retention of these health workers. Although these enablers and barriers have been reported from the included studies pertaining to nutrition integration into health systems, these are applicable to generic health system integration, as well. We have reported the information pertaining to the extent to which these were specific to nutrition integration, where such information was available. In Bangladesh, these barriers led to insufficient number of health facility staff to handle outpatient SAM and MAM caseloads in community management programmes, absence of a formal referral mechanism, reports of referred children receiving inadequate treatment at the hospital, and poor quality of care for CHW referrals sent (Kouam et al., 2014). Countries in sub‐Saharan Africa also reported increased staff workload, supervisory responsibilities, labour‐intensive tasks, and inadequate staff to implement the integrated nutrition‐specific activities (Deconinck et al., 2016). To respond to these barriers, programmes introduced new cadres of health care workers, so that the existing workers are not overburdened, whereas others introduced performance‐based incentives for existing workers to improve motivation and retention; however, the extent to which these approaches were successful was not evaluated. Supportive government policies and engagements were highlighted as important for sustainable progress.

**Table 3:** Key findings by building block

Building blocks	Findings	Enablers	Barriers
**Governance:** To what extent evidence‐based nutrition‐specific interventions were integrated into health policies and strategies	Most programmes consulted with stakeholders, and nutrition‐specific interventions were included in existing systems and strategies.	Strong health systems	Lack of stakeholder coordination
		District‐level evidence‐based planning and costing Resource mobilization driven by multisectoral development goals, and integrated assessment tools	
**Financing:** To what extent funds for nutrition‐specific interventions were allocated through domestic health funds	Most integrated nutrition‐specific interventions had external funding which did not come through existing health system financing.	Planning, budgeting and mobilizing with donors and other stakeholders Expenditure mapping at district level Funding distribution Community based financing Involving private sectors and contracting	Funding largely driven by development partners who continue to separate health and nutrition funding Lack of coordination in case of multiple funding sources Nutrition programme activities being stopped due to transition between funding cycles
**Information:** To what extent is the information on nutrition status and coverage of nutrition‐specific services integrated into existing information systems	Most programmes devised separate information system mechanisms for nutrition‐specific indicators.	Effective flow of information across the stakeholders and all levels of care Involvement of all major health actors Efforts to generate robust data and operational systems (using information technology) for intelligible and transparent collecting, tracking and reporting Use of robust data for identification of underserved population	Absence of nutrition indicators in the existing health information system
**Health workforce:** To what extent do the existing facility and community health workers offer nutrition‐specific services	Almost all programmes used existing facility‐ and community‐level staff to offer integrated nutrition‐specific services.	Hardship allowances for remote postings and supportive supervision visits including observation of case management. Workload management	Increased workload No CHW supervision and support Poor referral mechanisms Poor quality of care once referred
**Supplies/technology:** To what extent existing infrastructure and commodity supply were used for nutrition‐specific interventions	Though some programmes enhanced existing channels, others set up separate nutrition‐specific channels.	Effective logistics system for medicines and mass drug distribution Promoting in‐country drug manufacturers Appropriate equipment and maintenance	Instability of nutrition commodities like nutrition supplements Stock‐outs and wait times
**Service delivery:** To what extent do existing facility and CHW offer quality nutrition‐specific services	Most programmes offered integrated services through existing delivery mechanisms.	Co‐location of services Coordinated messages and increased motivation among health personnel	Inadequate training Absence of effective referral mechanism Increased workload due to addition of nutrition related services

*Note*. CHW: community health worker.

A majority of the programmes involved some level of consultation with government stakeholders and ministry staff to include nutrition‐specific interventions in existing health system and strategies. However, barriers hindering satisfactory integration of governance included lack of coordination across ministry of health directorates and lack of mechanisms for large‐scale motivation, supervision, and support to CHW. In Tanzania, implementation of an “IMCI supervision checklist” was not possible owing to the many duties that supervisors were expected to perform (Armstrong et al., 2004; Masanja et al., 2005), although lack of mechanisms for CHW motivation, supervision, and support on a large scale were also reported. Programmes from sub‐Saharan Africa and South Asia have also reported lack of coordination across ministry of health directorates and ineffective supervision given multiple and additional tasks necessitated by integration of activities (Rasanathan et al., 2014). There were no real examples of negotiated “task adjustments” and right‐sizing of supervisory and management tasks of integrated programmes, and in most instances, additional activities were generally additive.

There was also a lack of coordination among funding streams, and most funding for integrated nutrition‐specific interventions was through special projects and grants rather than from existing health systems financing mechanisms (Deconinck et al., 2016; El Arifeen et al., 2004; Rasanathan et al., 2014). In Bangladesh, complications related to the transition between two government funding cycles were reported that resulted in the nutrition programme activities being stopped (Puett et al., 2013). In sub‐Saharan Africa, funding was largely driven by development partners, even for aspects that would be expected to be covered by governments, such as salaries and commodities hampering sustainability of the programmes (Palmer et al., 2013). This is a key area for strengthening in future strategies for sustainability of integrated nutrition interventions, as separate streams are likely to remain separate and short‐lived. Communication and information flow across the continuum of care (including data rollup from the facility to the subnational and then national, levels) was a notable challenge, as ensuring inclusion of useful nutrition indicators in the health management information system was difficult because the existing programmes did not include nutrition‐specific indicators, and most of the programmes devised separate mechanisms to gather data on nutrition‐specific indicators, which also hampered integrated management, oversight, and accountability. Commodities, medicines, and related supply chain were poorly integrated as some programmes set up a separate channel for nutrition‐specific interventions' supplies, although others enhanced the existing setups for timely supply. Because drug shortages were reported as a major challenge in several programmes, as in India, stability of the nutrition products was also reported as one of the challenges (Taneja et al., 2015). Countries from sub‐Saharan and South Asia reported irregular medicine supply, including nutrition supplements, stock‐outs, and wait times for supplies (Doherty et al., 2010; Palmer et al., 2013). Such shortages not only delay implementation but also impact demand and service delivery. In Bangladesh, irregular supplies of medicines from the health facilities were reported to lead to diminution of the community members' trust in CHW and the programme (Kouam et al., 2014).

## 5 CONCLUSIONS

The scarce data around integrated nutrition programmes reveal mixed evidence and information gaps. The evidence does suggest, however, that there is much potential for integrating nutrition interventions into health and related programmes to ensure adequate, efficient service delivery, and impact on nutrition and non‐nutrition outcomes. There are some examples of well‐integrated programmes, yet evidence also underscores the need for more well‐planned and well‐designed programmes, taking into account all the building blocks to ensure efficiency, long‐term sustainability, and impact.

## CONFLICTS OF INTEREST

The authors declare that they have no conflicts of interest.

## CONTRIBUTIONS

RAS, JKD, and ZAB designed the conceptual framework and tools. RAS and JKD conducted the search, extraction and the analysis. All authors have read and approved the final manuscript.

## Supporting information

Data S1 Supporting Information
