# Supplementary material for: Integrating nutrition into health systems: What the evidence advocates
Source: Matern Child Nutr. 2019 Feb 12;15(Suppl 1):e12738. doi: 10.1111/mcn.12738 (PMC6594109; doi:10.1111/mcn.12738)
Supplement: Supplementary file 1 — Data S1 Supporting Information [file MCN-15-e12738-s001.docx]

**Annex 1: Conceptual Framework to guide the Scoping Review**


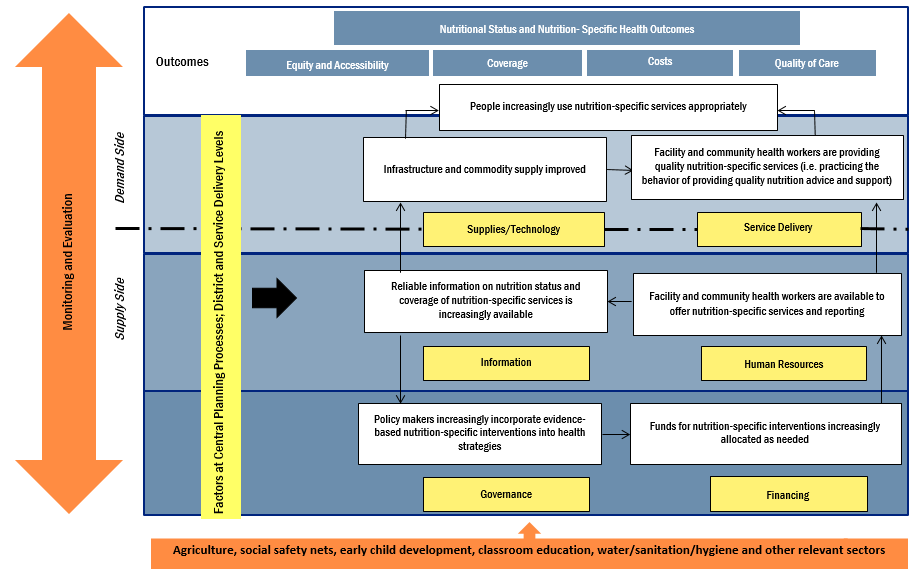


**Annex 2: Study selection and risk of Bias**

**Figure 1: Search flow diagram**


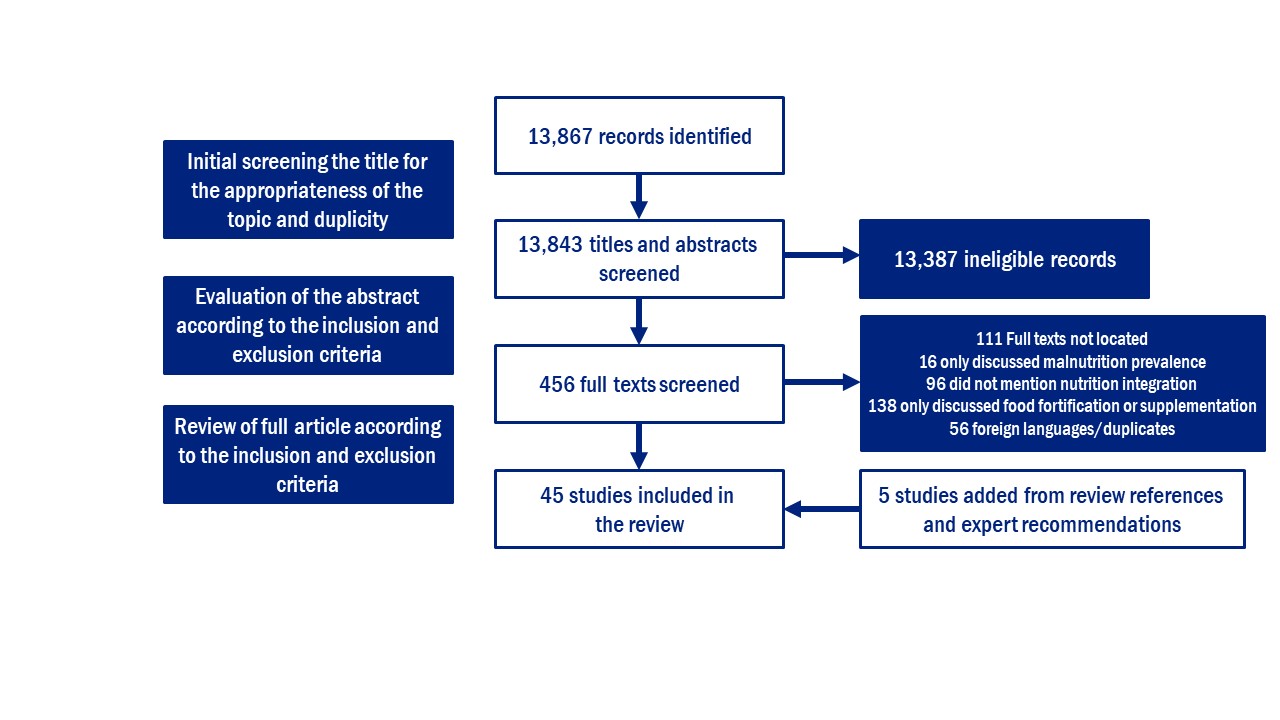


Table 1: Summary of studies and nutrition interventions included

| **Integration** | **Studies & Programs** | **Study Design** | **Locations** | **Nutrition Interventions Included** |
| --- | --- | --- | --- | --- |
| **Nutrition into IMCI/iCCM** | 12 studies from 6 programs (Arifeen et al., 2009; Armstrong et al., 2004; Bhandari et al., 2012; Bryce et al., 2005; El Arifeen et al., 2004; Friedman & Wolfheim, 2014; Masanja et al., 2005; Mazumder et al., 2014; Miller et al., 2014; Rasanathan et al., 2014; Schellenberg et al., 2004; Taneja et al., 2015) | Five were randomised controlled trials (RCTs), five were nonrandomised comparisons, one was a cross-sectional survey and one was a qualitative study. | Bangladesh, India, Tanzania, Ethiopia; 45 countries in sub-Saharan Africa | Counselling of mothers on breastfeeding and appropriate complementary feeding, local feeding practices, growth monitoring, supplementary nutrition, vitamin A supplementation, and screening, management and referral for malnutrition. |
| **SAM/MAM into Health Services** | 10 studies from 6 programs (Aguayo et al., 2013; Amadi et al., 2016; Brits et al., 2017; Deconinck et al., 2016; Kouam et al., 2014; Puett et al., 2015; Puett et al., 2013; Sadler et al., 2011; Tadesse et al., 2017) | Four were cohort, one was a cross-sectional survey, three were qualitative, one was of mixed design, and one was a cost-effectiveness study. | Niger, Bangladesh, India, Ethiopia, South Africa and Zambia | Community and facility-based management of SAM and MAM. |
| **Nutrition into Child Health Days** | 2 studies from 2 programs (Doherty et al., 2010; Palmer et al., 2013) | Both studies were qualitative assessment of the programs. | Countries from sub-Saharan Africa and southern Asia | Vitamin A supplementation and nutrition screening. |
| **Nutrition into Immunization** | 6 studies from 6 programs (Anand et al., 2012; Baqui et al., 2008; Ching et al., 2000; Hodges et al., 2015; Klemm et al., 1996; Ropero-Álvarez et al., 2012) | Three were quasi-experimental and three were secondary data analysis of the existing survey datasets. | Philippines, Vietnam, Sierra Leone, sub-Saharan Africa, Latin America and Caribbean | Vitamin A supplementation, early and exclusive breastfeeding, infant and young child feeding practices and growth monitoring. |
| **Nutrition into ECD** | 3 studies from 2 programs (Fernandez‐Rao et al., 2014; Gowani et al., 2014; Yousafzai et al., 2014) | All three were RCTs. | India and Pakistan | Home/preschool fortification with multiple micronutrient powder, responsive stimulation, early nutrition interventions, monitoring of child nutrition and growth promotion. |
| **Nutrition into Cash Transfer Programs** | 1 study from 1 program (Grellety et al., 2017) | The study was an RCT. | Congo | Treatment of SAM according to the national protocol and counselling with or without a cash supplement of US$40 monthly for 6 months. |
| **Nutrition into Other Programs** | 11 studies from 8 programs (Berti et al., 2010; Fagerli et al., 2017; Grossmann et al., 2015; Guyon et al., 2009; Nguyen et al., 2017; Parikh et al., 2010; Saiyed & Seshadri, 2000; Singh et al., 2017; Sivanesan et al., 2016; Tandon, 1989) | Six were quasi-experimental, one was RCT, two were before and after studies and two were cross-sectional surveys. | Various | Infant and young child feeding practices and micronutrient supplementation. |

**Figure 2: Risk of Bias of the Included Studies**

**
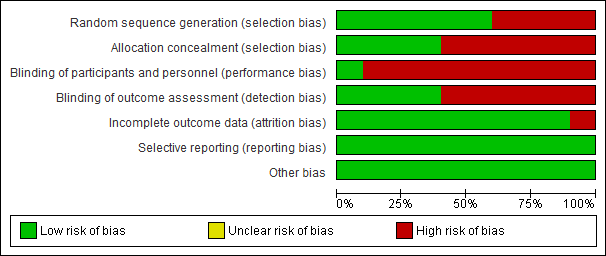
**

**
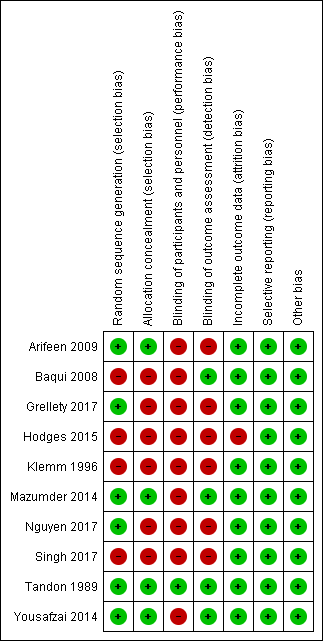
**
